# Diastereoselective Axial-to-Chelate-Puckering Chirality Transfer in *cis*-[PtCl_2_(DABN)] Complexes

**DOI:** 10.1021/acs.inorgchem.6c03354

**Published:** 2026-09-09

**Authors:** Andrea Maluenda, Tamara Bolaño, Pablo J. Sanz Miguel

**Affiliations:** † Departamento de Química Inorgánica, Instituto de Síntesis Química y Catálisis Homogénea (ISQCH), Universidad de Zaragoza-CSIC, 50009 Zaragoza, Spain; ‡ Departamento de Química Inorgánica, Instituto de Nanociencia y Materiales de Aragón (INMA), Universidad de Zaragoza-CSIC, 50009 Zaragoza, Spain

## Abstract

Axially chiral 1,1′-binaphthalene-2,2′-diamine (DABN) was used to control chelate-ring puckering in *cis*-[PtCl_2_(DABN)] complexes. Coordination of *R*_a_- or *S*_a_-DABN affords the enantiomeric complexes *cis*-λ-[PtCl_2_(*R*_*a*_-DABN)] (λ,*R*_*a*_-**1**) and *cis*-δ-[PtCl_2_(*S*_*a*_-DABN)] (δ,*S*_*a*_-**1**), respectively, with no evidence for mismatched diastereomers. X-ray crystallography shows that axial ligand chirality is translated into a configurationally locked λ or δ pucker of the seven-membered metallacycle. NMR spectroscopy supports retention of the structure in CD_3_CN, whereas kinetic studies in DMSO-*d*_6_ reveal pseudo-first-order DABN displacement by solvent. The enantiomeric complexes display intense mirror-image CD spectra, highlighting DABN as an effective axial chirality source for axial-to-chelate-puckering chirality transfer.

## Graphical abstract

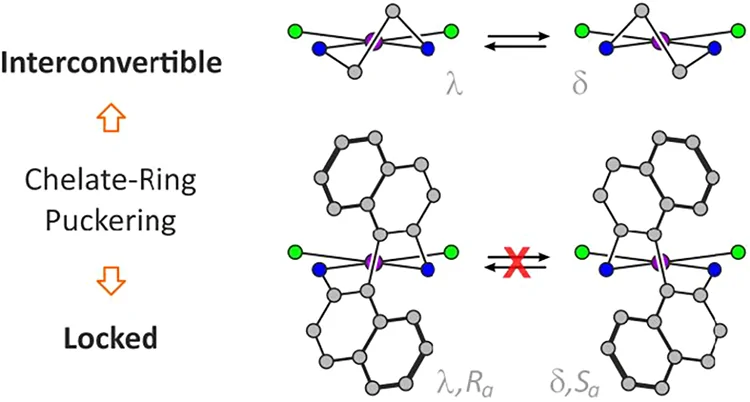

## Introduction

Square-planar Pt^II^ complexes of the general type *cis*-[PtCl_2_N_2_], with mutually *cis* N-donor ligands and *cis* disposed chlorido ions, constitute a well-established family of compounds historically exemplified by cisplatin, *cis*-[PtCl_2_(NH_3_)_2_].^1,2^ Beyond its biological relevance, cisplatin has inspired a broad coordination chemistry based on platinum complexes bearing N-donor ligands such as ammines,^3^ simple amines,^4,5^ N-heterocycles,^6–11^ nucleobases,^12,13^ or chelating diamines.^14–17^ Many of these systems are useful models for investigating situations as ligand substitution,^18,19^ *trans*-effect reactivity,^20–22^ metal-migration events,^23–26^ or supramolecular organization.^27–29^

In this context, *cis*-chelated Pt^II^ complexes are particularly interesting for examining how ligand nature influences molecular conformation and stereochemistry,^30,31^ since their stereochemical properties may reflect ligand-based stereogenic elements, metal-centered chirality,^32–34^ or the conformation of the chelate ring.^35–39^ For five-membered ethylenediamine-type chelate rings, puckering is typically described in terms of λ and δ conformations, following the nomenclature of Corey and Bailar.^40^ However, these conformations are often flexible and interconvert in solution. Recently, we have explored unconventional expressions of chirality, including chiral-at-metal systems,^41,42^ and solvent-induced chirality.^43^ The present study extends this interest to a different intramolecular scenario, in which axial ligand chirality is translated into a fixed chelate-ring conformation.

A suitable strategy to retain conformational chirality is to use a chiral ligand framework capable of enforcing a preferred chelate-ring pucker. This may be viewed from the perspective of ligand-to-metal chirality transfer,^44,45^ but with chirality expressed through chelate-ring conformation rather than through the generation of a stereogenic metal center.

Following this idea, we focused on axially chiral binaphthyl ligands, as their rigid atropisomeric skeletons can impose spatial constraints upon coordination. Among this family, BINOL, 1,1′-binaphthalene-2,2′-diol, has played a pioneering role in asymmetric catalysis and chiral recognition,^46^ and structural modification of the binaphthyl scaffold has led to widely used ligands and catalysts.^47,48^ In contrast, the less explored diamino analogues allow direct access to metal coordination via *N*,*N*-chelation.^49^ Thus, incorporation of the axially chiral diamine 1,1′-binaphthalene-2,2′-diamine (DABN) into the *cis*-[PtCl_2_N_2_] skeleton provides an opportunity to examine how axial ligand chirality can be translated into a defined chelate-ring conformation within the metal coordination sphere.

Herein, we report the diastereoselective synthesis of the enantiomeric complexes *cis*-λ-[PtCl_2_(*R*_*a*_-DABN)] (λ,*R*_*a*_-**1**) and *cis*-δ-[PtCl_2_(*S*_*a*_-DABN)] (δ,*S*_*a*_-**1**), with no evidence for the corresponding mismatched diastereomeric combinations. Their structural, solution, and chiroptical properties are examined in the context of axial-to-chelate-puckering chirality transfer in square-planar complexes.

## Results and Discussion

### Solid State Structure and Stereochemical Analysis

Treatment of K_2_[PtCl_4_] with enantiopure *R*_*a*_-DABN or *S*_*a*_-DABN in acetonitrile afforded the corresponding enantiomeric complexes λ,*R*_*a*_-**1** or δ,*S*_*a*_-**1**, respectively, in a diastereoselective manner (see below for the chirality descriptors). Single crystals suitable for X-ray diffraction were grown by slow diffusion of diethyl ether into acetonitrile solutions of the enantiopure complexes at 4 °C. Both λ,*R*_*a*_-**1** and δ,*S*_*a*_-**1** crystallized in the chiral space group *C2* and exhibit closely similar unit-cell parameters, consistent with their enantiomeric nature.

Views of the molecular structures of λ,*R*_*a*_-**1** and δ,*S*_*a*_-**1** are depicted in Figure 1. Both complexes exhibit equivalent geometrical features and differ only in the chirality of the DABN ligands and the associated chelate-ring pucker. Therefore, only λ,*R*_*a*_-**1** is further discussed in detail. Its Pt center adopts a slightly distorted square-planar coordination geometry defined by two amine groups from the DABN ligand and two chlorido ions. The Pt-coordination plane is essentially planar, with the Pt atom displaced by 0.004(2) Å from the least-squares plane defined by the four donor atoms. Pt–N and Pt–Cl bond lengths are within the expected values: Pt1–N2a, 2.051(6) Å; Pt1–N2b, 2.069(5) Å; Pt1–Cl1, 2.2934(17) Å; Pt1–Cl2, 2.3001(17) Å. The *cis* angles around Pt, ranging from 86.16(15)° (N2a–Pt1–Cl2) to 94.27(7)° (Cl1–Pt1–Cl2), and the *trans* angles of 177.90(18)° (N2a–Pt1–Cl1) and 176.32(15)° (N2b–Pt1–Cl2) further support the square-planar arrangement.

**Figure 1:**
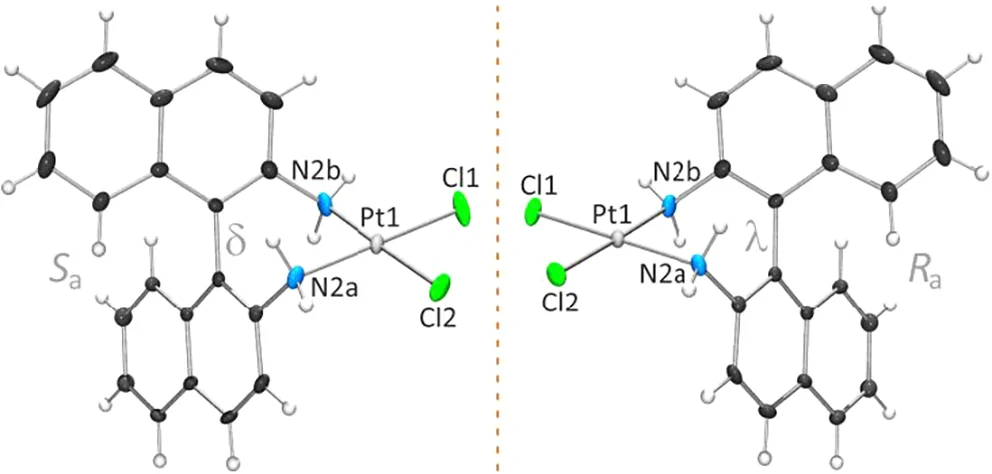
View of enantiomers δ,*S*_*a*_-**1** and λ,*R*_*a*_-**1**. Thermal ellipsoids are drawn at the 40% probability level. Flack parameters are −0.016(3) for δ,*S*_*a*_-**1** and −0.009(3) for λ,*R*_*a*_-**1**.

In the crystal packing of λ,*R*_*a*_-**1**, short N2a­(H)···Cl2 contacts, 3.181(6) Å, connect each complex to two symmetry-related neighbors, generating a supramolecular *M*-helical motif along the *b* axis (Figure 2). As expected, δ,*S*_*a*_-**1** forms the enantiomeric *P*-helical arrangement, for which the *b* axis repeat corresponds to a full 360° rotation and defines the helical pitch, 7.6646(3) Å. Weak contacts involving the naphthalene rings provide additional lattice stabilization, although no significant π–π stacking is observed.

**Figure 2:**
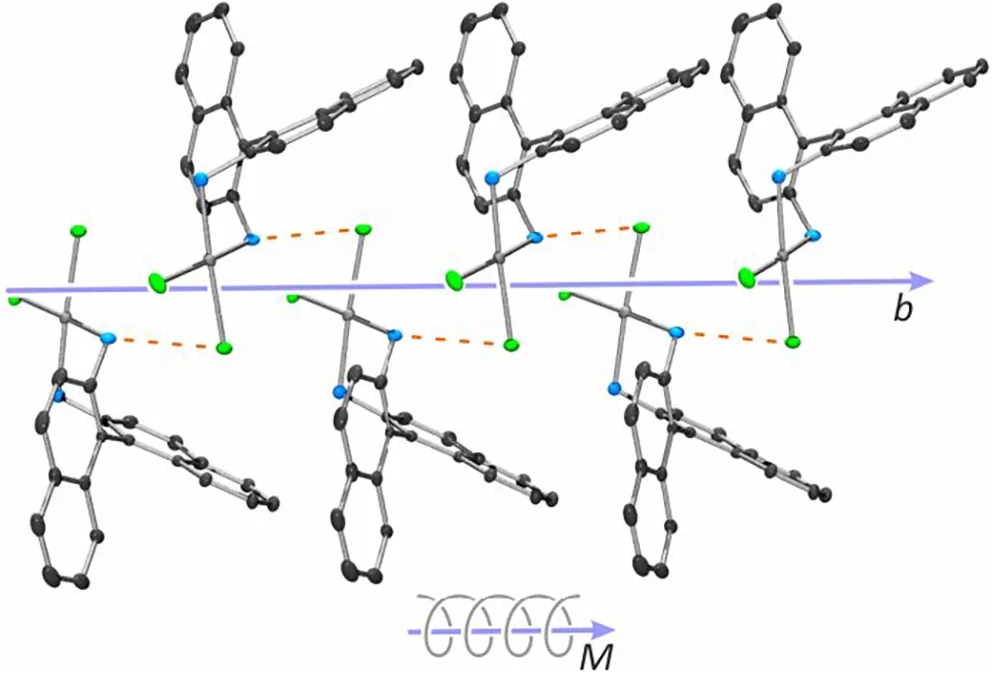
Detail of the N–H···Cl hydrogen-bonding motif in λ,*R*_*a*_-**1**, highlighting the *M*-helical motif along the *b* axis.

Chelation of DABN generates a seven-membered metallacycle adopting an approximately *C*_2_-symmetric twist conformation. The N2a–Pt–N2b bite angle in λ,*R*_*a*_-**1** is 90.57(19)°, indicating that DABN is accommodated within the square-planar coordination sphere without imposing significant strains. As expected, DABN retains its atropisomeric geometry upon coordination, with a dihedral angle of 63.44(12)° between the two naphthyl main planes. This value indicates a slightly less pronounced twist of the binaphthyl backbone than that observed in the free ligand (68.2°),^50^ while remaining within the range of related square-planar *cis*-M­(DABN) complexes.^51–53^

As mentioned above, five-membered metallacycles may adopt λ or δ puckered conformations, as the en-chelate ring in *cis*-[PtCl_2_(en)] (en = ethylenediamine), for which δ ⇄ λ inversion barriers of ca. 20 kcal mol^–1^ have been estimated.^54,55^ Using this notation by analogy, the seven-membered Pt-metallacycle in λ,*R*_*a*_-**1** can be described as adopting a λ-helical pucker, whereas the *S*_*a*_-DABN analogue displays the corresponding δ configuration (Figure 3). Unlike flexible five-membered en-chelate rings, these puckers are configurationally locked by the rigid axial chirality of DABN, and δ ⇄ λ interconversion would require disruption of metal coordination and further inversion of the ligand axial configuration.

**Figure 3:**
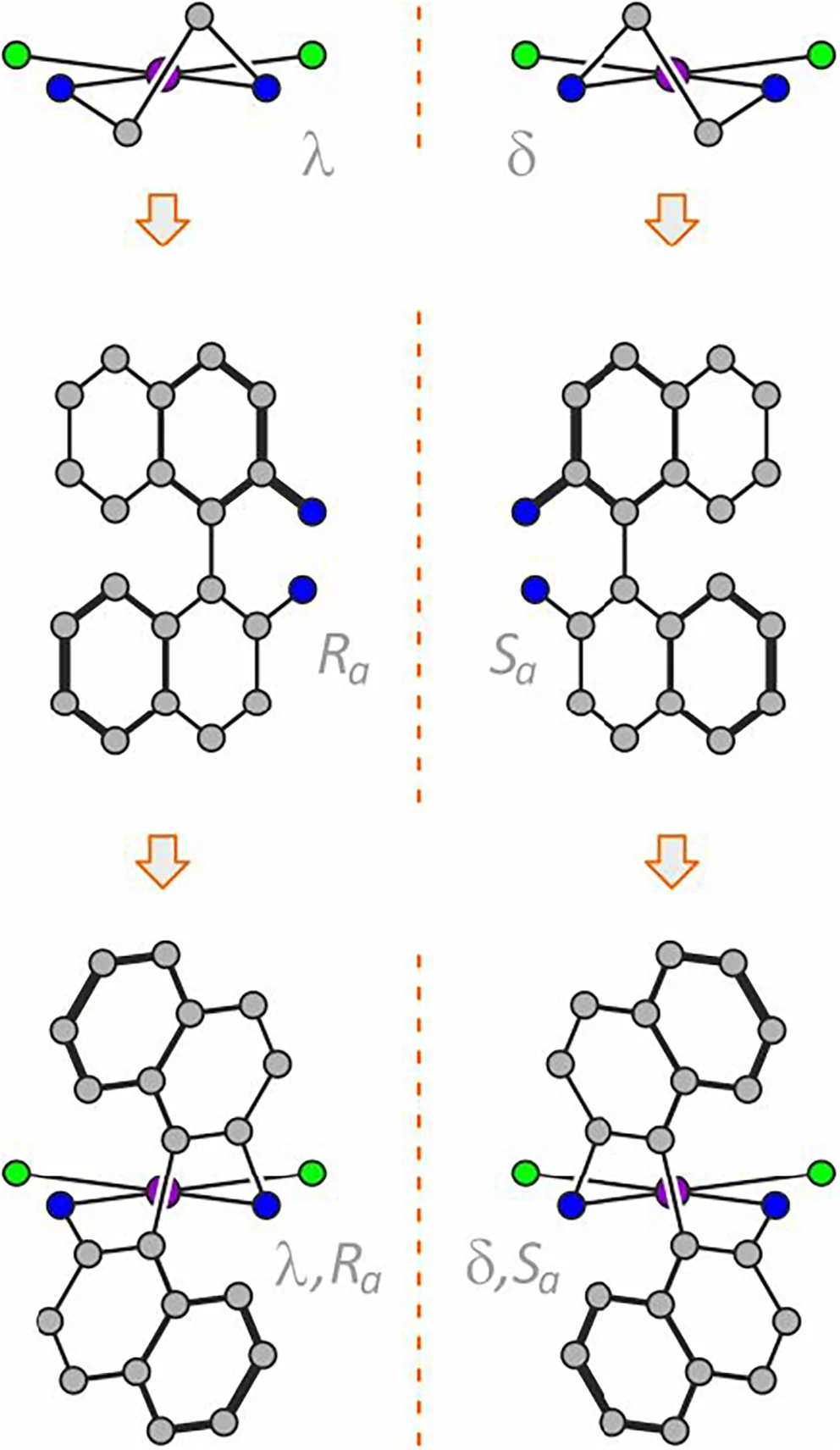
Conceptual illustration of λ/δ puckering descriptors. Top: five-membered en-chelate ring in *cis*-[PtCl_2_(en)] viewed along the *C*_2_ axis. Middle: axially chiral *R*_*a*_- and *S*_*a*_-DABN ligands. Bottom: seven-membered Pt-chelate rings in λ,*R*_*a*_-**1** and δ,*S*_*a*_-**1**, assigned by analogy. Cl, green; N, blue; C, gray; Pt, violet.

No evidence for the mismatched diastereomeric combinations, namely δ,*R*_*a*_-**1** and λ,*S*_*a*_-**1**, was found by X-ray crystallography or NMR spectroscopy. The matched chelate-ring puckers accommodate the binaphthyl framework, whereas the alternative puckers would be expected to require greater closure of the inter-naphthyl dihedral angle and less favorable contacts between rings. The present data do not allow us to distinguish whether this selectivity is kinetic or thermodynamic. Interconversion to alternative chelate-ring configurations would require substantial conformational reorganization and may involve Pt–N bond dissociation/recoordination, while any pathway involving inversion of the DABN axial configuration would additionally suffer the atropisomerization barrier of the ligand. Thus, the axial configuration of DABN favors the chelate-ring pucker that preserves a favorable binaphthyl geometry.

We next examined crystallization from a 1:1 racemic mixture of λ,*R*_*a*_-**1** and δ,*S*_*a*_-**1**. Under the same low-temperature vapor-diffusion conditions used for the homochiral samples, both enantiomers crystallized together as a racemic compound, rac-**1**, in the centrosymmetric space group *P2*_1_/*c*, with the opposite enantiomer generated by inversion symmetry. The molecular geometry of rac-**1** is similar to that observed in the enantiopure *C*_2_-crystals. Pt–N and Pt–Cl bond lengths, and coordination angles around the platinum center are not significantly different from those observed in λ,*R*_*a*_-**1** and δ,*S*_*a*_-**1** (Supporting Information). The main intramolecular difference is found in the axial twist of the DABN scaffold, which increases to 67.52(6)°, ca. 4° wider than in the enantiopure crystals. Thus, the main differences between the homochiral and racemic solids arise from crystal packing rather than from significant changes in molecular geometry.

The packing of rac-**1** is likewise governed by N–H···Cl contacts, although their organization differs markedly from that in the enantiopure crystals. Two symmetry-related pairs of contacts connect each complex to neighboring complexes of opposite chirality: a pair at 3.237(4) Å and a second, slightly longer pair at 3.344(4) Å on the opposite side (Figure 4). These interactions propagate along the *a* axis, and generate an alternating up-down supramolecular motif rather than the *M*-helical arrangement observed in λ,*R*_*a*_-**1**. Although individual N–H···Cl contacts in rac-**1** are slightly longer than those observed in λ,*R*_*a*_-**1**, their arrangement as two pairs of interactions results in a larger number of hydrogen bonding contacts between adjacent molecules.

**Figure 4:**
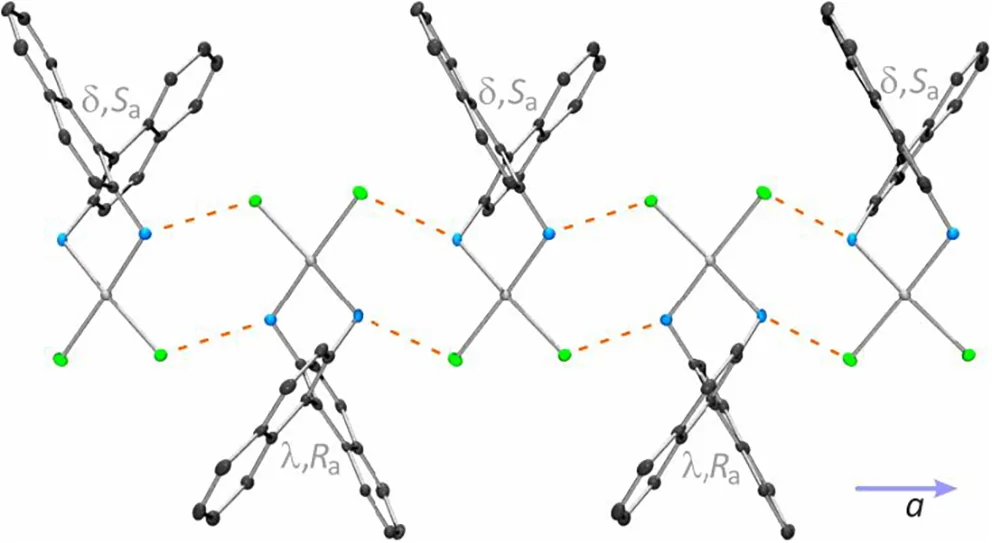
Detail of the twofold N–H···Cl hydrogen-bonding pattern in rac-**1** along the *a* axis.

Consistent with this interpretation, rac-**1** displays a higher density (2.128 g cm^–3^) than the corresponding homochiral forms (λ,*R*_*a*_-**1**, 2.038 g cm^–3^; δ,*S*_*a*_-**1**, 2.028 g cm^–3^), representing a 4.7% increase relative to their average density. This behavior is in line with Wallach’s rule,^56^ which states that racemic crystals often pack more efficiently than enantiopure crystals. Thus, combination of the larger number of N–H···Cl contacts and improved enantiomeric complementarity seems to favor formation of the centrosymmetric racemic compound rather than the separate crystallization of λ,*R*_*a*_-**1** and δ,*S*_*a*_-**1**.

### NMR Analysis

NMR spectra of λ,*R*_*a*_-**1** and δ,*S*_*a*_-**1** recorded in CD_3_CN at room temperature are consistent with the coordination geometry revealed by X-ray crystallography. As expected for enantiomers in an achiral medium, both complexes display identical chemical shifts and coupling constants.

The ^1^H NMR spectrum of λ,*R*_*a*_-**1** displays a *C*_2_-symmetric pattern comprising seven resonances (Figure 5). Six of them appear in the aromatic region, between *δ* 8.16 and 7.03 ppm, and are assigned to the naphthyl protons of DABN. Four doublets at δ 8.16, 8.06, 7.58, and 7.03 ppm correspond to H4, H5, H3, and H8, respectively, while the triplets at δ 7.52 and 7.35 ppm are assigned to H6 and H7. Comparison with free DABN (Supporting Information) reveals overall downfield shift and a simplification of the aromatic pattern upon coordination. The characteristic multiplets and long-range couplings observed for the free ligand are replaced by better-resolved, ortho-coupled resonances in λ,*R*_*a*_-**1**. Interestingly, the NH_2_ region displays two partially resolved components of an AB-type pattern between δ 5.85 and 5.54 ppm, consistent with diastereotopic N–H protons in the rigid, puckered chiral environment generated upon chelation. Weak ^195^Pt satellites are also discernible within this resonance, further supporting Pt–N coordination.

**Figure 5:**
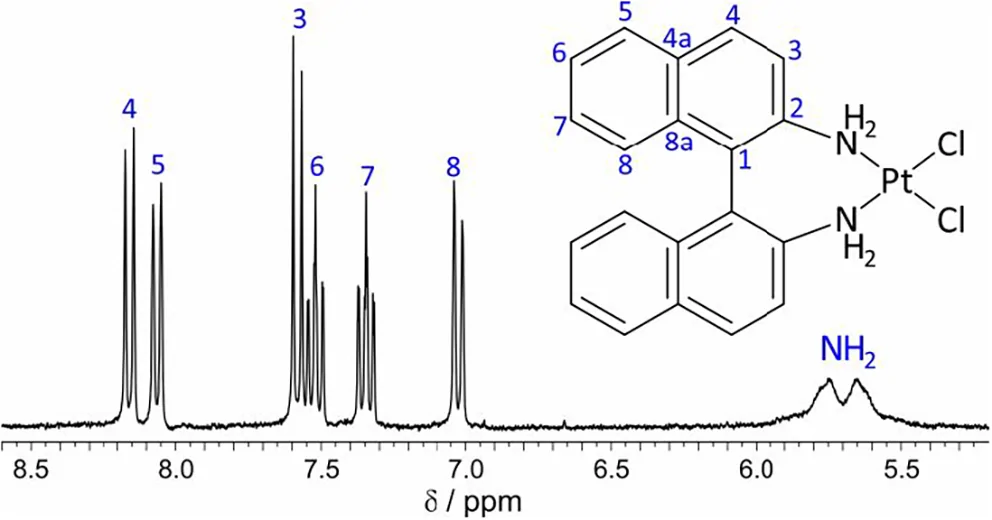
^1^H NMR spectrum of λ,*R*_*a*_-**1** (CD_3_CN, r.t.), including atom numbering scheme.

Variable-temperature ^1^H NMR spectra in CD_3_CN (223 –343 K, 500 MHz) show essentially unchanged C­(H) resonances, while the N­(H) signals undergo moderate temperature-dependent shifts and broadening, with better resolution of the AB pattern at low temperature (Supporting Information). No additional resonances or significant changes attributable to inversion of the seven-membered chelate ring are observed.

The ^13^C­{^1^H} NMR spectrum shows ten resonances in the aromatic region, as expected for the ten symmetry-inequivalent carbon environments of the *C*_2_-symmetric DABN ligand. Four resonances at *δ* 138.5, 134.7, 133.2, and 122.0 ppm are assigned to quaternary carbon atoms C2, C8, C4A, and C1, respectively. The remaining six resonances at *δ* 132.2, 129.5, 128.3, 126.8, 126.3, and 121.1 ppm correspond to the aromatic CH carbons C4, C5, C7, C6, C8, and C3, respectively.

Finally, the ^195^Pt­{^1^H} NMR spectrum exhibits a single resonance at δ −1914.4 ppm, consistent with a square-planar platinum center coordinated by two chlorido ions and two amine donor atoms from the DABN ligand.^57^

### DABN Displacement in DMSO Solution

The solution stability of δ,*S*_*a*_-**1** was examined in DMSO-*d*_6_ by time-dependent ^1^H NMR spectroscopy. Upon dissolution, δ,*S*_*a*_-**1**, undergoes progressive displacement of the chelating DABN ligand by solvent molecules, giving free DABN together with a Pt–DMSO species, consistent with the known affinity of platinum for DMSO. No intermediates or alternative Pt–DABN species were detected by ^1^H NMR spectroscopy. Owed to the clean evolution of the process, a kinetic study was carried out.

Evolution of δ,*S*_*a*_-**1** in DMSO-*d*_6_ was followed at room temperature by recording consecutive ^1^H NMR spectra (Figure 6). This process was quantified by integration of non-overlapping resonances H6 and H3 corresponding to coordinated and free *S*_*a*_-DABN. Resulting concentration profiles are shown in Figure 7.

**Figure 6:**
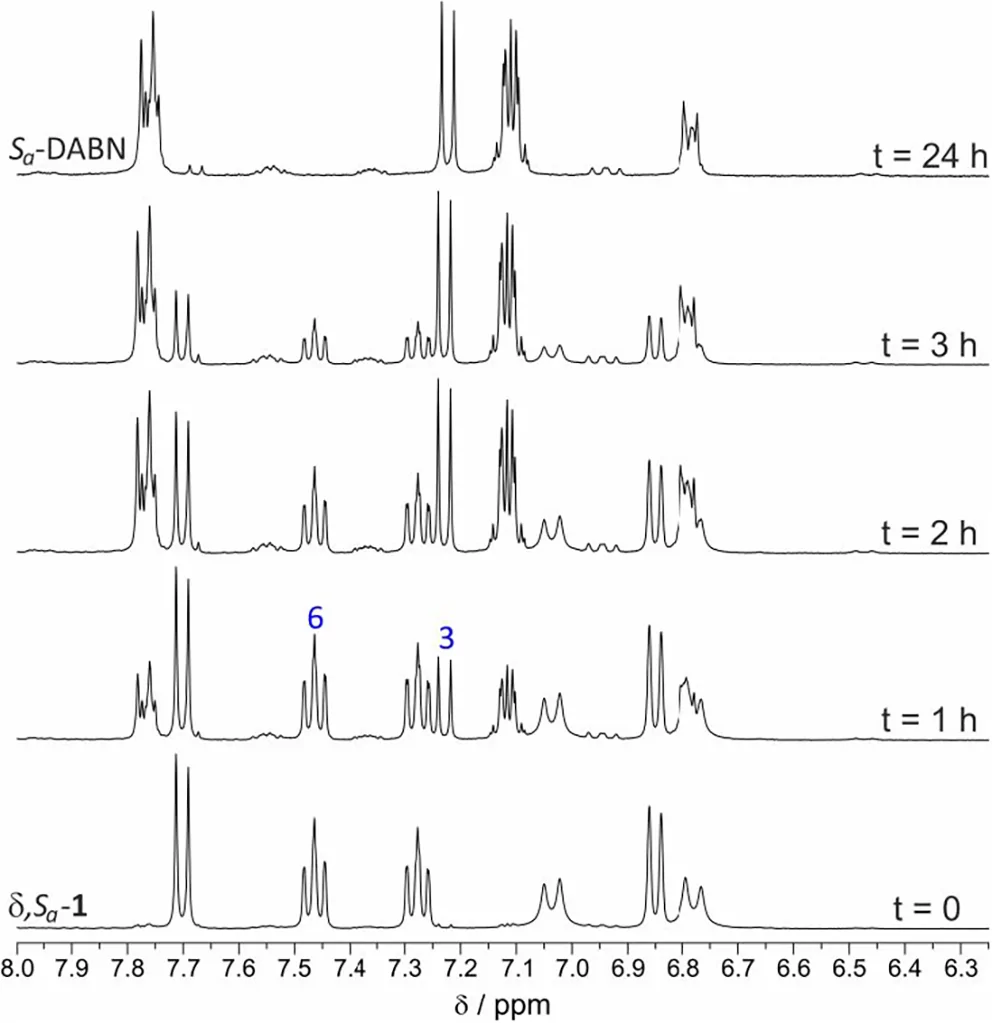
Time-dependent ^1^H NMR monitoring of δ,*S*_*a*_-**1** (DMSO-*d*_6_, r.t.), showing its progressive disappearance and release of free *S*_*a*_-DABN. Signal H6 is diagnostic of δ,*S*_*a*_-**1**, whereas signal H3 corresponds to free *S*_*a*_-DABN.

**Figure 7:**
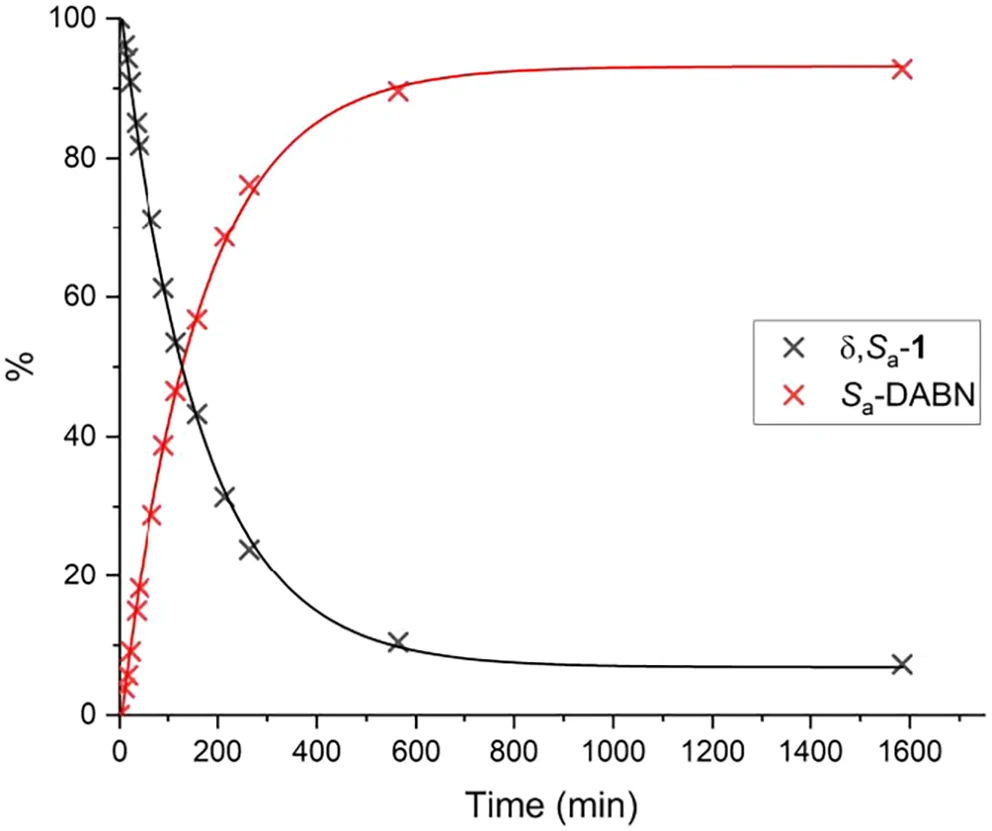
Evolution of δ,*S*_*a*_-**1** and *S*_*a*_-DABN in DMSO-*d*_6_. Solid lines correspond to pseudo-first-order fits using all 44 ^1^H NMR spectra. Selected data points are displayed for clarity.

Under these conditions, DMSO is present in a large excess and its concentration can be considered constant. Assuming overall replacement of DABN by DMSO, namely, *cis*-[PtCl_2_(DABN)] + 2 DMSO → *cis*-[PtCl_2_(DMSO)_2_] + DABN, the ligand-displacement process was therefore treated as a pseudo-first-order with respect to δ,*S*_*a*_-**1**, namely −d­[δ,*S*_*a*_-**1**]/dt = *k*_obs_[δ,*S*_*a*_-**1**]. Consequently, concentration of δ,*S*_*a*_-**1** decays exponentially with time, whereas the concentration of free DABN increases according to a complementarity exponential function (Supporting Information).

Fitting the disappearance of δ,*S*_*a*_-**1** and the appearance of free DABN afforded *k*_obs_ = 6.36×10^–3^ min^–1^, corresponding to *t*_1/2_ = 109 min. The quality of the fits, with *R* ^2^ = 0.99923 and 0.99926, respectively, supports a pseudo-first-order description. The exponential behavior is consistent with a DMSO-induced solvolysis process with no detectable intermediates on the NMR time scale.

Although kinetic data do not establish the substitution mechanism, they are compatible with a rate-limiting step involving Pt–N bond cleavage followed by rapid coordination of DMSO. The observed half-life indicates that δ,*S*_*a*_-**1** is moderately stable in DMSO solution. After 6 h, ligand displacement was nearly complete, with a coordinated/free DABN ratio of 8.7:91.3. This transformation was also evident visually, as the initially yellow solution gradually became colorless during the experiment.

### Circular Dichroism Analysis

Circular dichroism (CD) spectroscopy was used to compare the chiroptical responses of enantiopure and racemic DABN with those of the corresponding platinum complexes in acetonitrile. The two atropisomers of the free ligand, *R*_*a*_-DABN and *S*_*a*_-DABN, were first analyzed as references. Both enantiomers display Cotton effects centered at 376 and 310 nm, with opposite signs depending on the axial configuration of the ligand (Figure 8). Considering the conjugated aromatic structure of DABN, these features are tentatively attributed to ligand-centered π→π* transitions. This mirror-image relationship is consistent with their enantiomeric nature. As expected, rac-DABN is CD silent within experimental error, owing to cancellation of the equal and opposite chiroptical responses of both enantiomers (Supporting Information).

**Figure 8:**
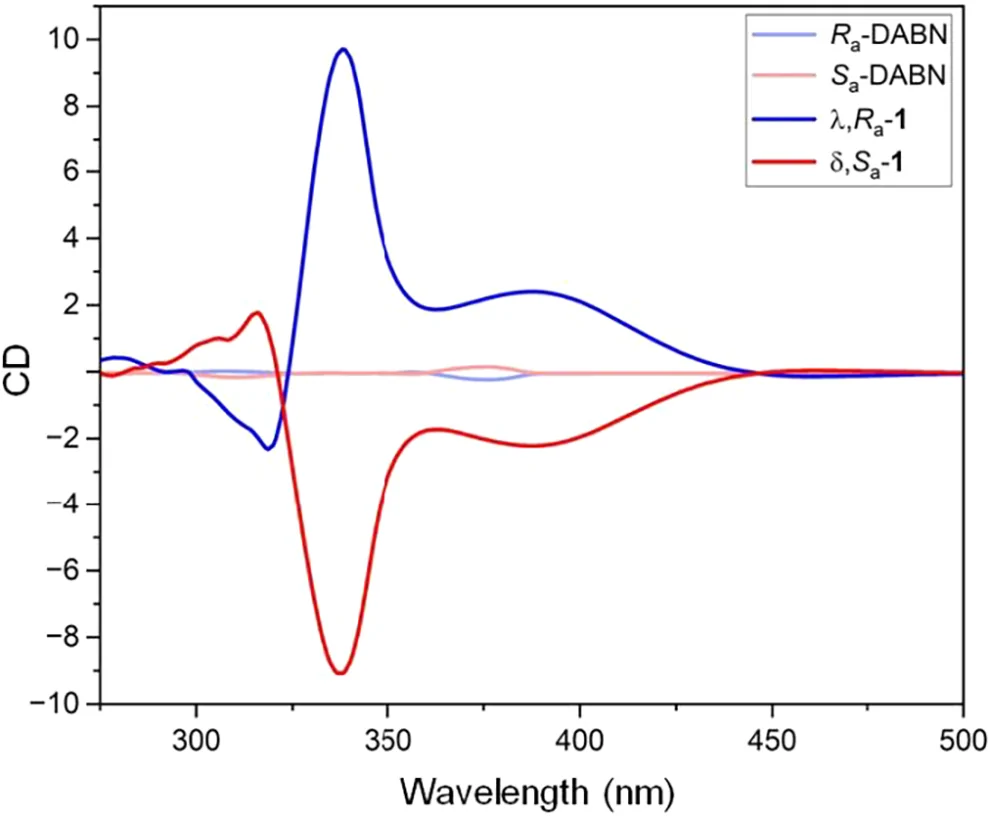
CD spectra of *R*_*a*_-DABN, *S*_*a*_-DABN, λ,*R*_*a*_-**1** and δ,*S*_*a*_-**1**, recorded in acetonitrile at room temperature. The *Y*-axis is ellipticity *θ* (10^6^ deg·cm^2^/dmol).

The CD spectra of λ,*R*_*a*_-**1** and δ,*S*_*a*_-**1**, were then recorded under the same conditions. They exhibit substantially more intense Cotton effects than the free ligand, with bands at 388, 338, and 318 nm (Figure 8). These bands are also assigned primarily to ligand-centered transitions perturbed by platinum coordination. Both spectra are mirror images of each other, consistent with their enantiomeric nature and with the opposite axial configurations and chelate-ring helicities. In contrast, rac-**1** does not display a measurable CD signal, consistent with cancellation of responses of the two enantiomers (Supporting Information).

Comparison of the CD spectra of free DABN and the platinum complexes reveals changes in both band position and intensity upon coordination, although do not allow the individual contributions of the ligand axial chirality and the chelate-ring helicity to be separated. In particular, coordination to the metal ion enhances the CD response and shifts the main Cotton effects, which is consistent with rigidification of the binaphthyl scaffold and modification of its electronic structure upon chelation. These changes provide further evidence for the formation of λ,*R*_*a*_-**1** and δ,*S*_*a*_-**1** and support the configurational assignments derived from X-ray crystallography.

## Conclusions

Enantiopure *R*_*a*_-DABN and *S*_*a*_-DABN enable the diastereoselective synthesis of the enantiomeric complexes λ,*R*_*a*_-**1** and δ,*S*_*a*_-**1**, whereas no evidence for the mismatched combinations δ,*R*_*a*_-**1** and λ,*S*_*a*_-**1** was found. X-ray diffraction reveals that coordination of the axially chiral diamine generates a seven-membered chelate ring in which the ligand atropisomerism determines the λ or δ puckering of the Pt-metallacycle. This supports an axial-to-chelate-puckering chirality transfer process, leading to configurationally locked square-planar complexes.

The solid state structures reveal different packing preferences for the homochiral and racemic forms, with rac-**1** adopting a denser centrosymmetric lattice consistent with Wallach’s rule. NMR spectroscopy supports retention of the complex structure in CD_3_CN and reveals diastereotopic N–H protons associated with the rigidity of the chiral chelate. In DMSO-*d*_6_, a solvent-induced DABN displacement following pseudo-first-order kinetics is observed. CD spectroscopy further supports the configurational assignments, with the enantiomeric complexes displaying intense mirror-image Cotton effects that are enhanced compared to the free ligands.

Thus, DABN efficiently translates its axial chirality into a single, configurationally locked chelate-ring pucker, with no evidence for δ ⇄ λ interconversion. By expressing ligand chirality through chelate-ring conformation rather than through metal-centered stereogenicity, these complexes represent a distinct stereochemical model for axial-to-chelate-puckering chirality transfer in square-planar systems, with potential extension to octahedral analogues.

## Experimental Section

### General Procedures

All reagents and solvents were purchased from commercial sources and used as received. Glassware was dried at 120 °C before use. All reactions were carried out under aerobic conditions. NMR spectra were recorded on Bruker Avance 300, Bruker Avance 400, and Bruker Avance 500 spectrometers. NMR chemical shifts are reported in parts per million (ppm). ^1^H and ^13^C­{^1^H} NMR spectra were referenced to residual solvent resonances, whereas ^195^Pt NMR chemical shifts were referenced indirectly using the standard frequency calibration of the spectrometer. Coupling constants, *J*, are given in hertz (Hz). High-resolution electrospray ionization mass spectra (HRMS-ESI) were acquired using a MicroTOF-Q hybrid quadrupole time-of-flight spectrometer (Bruker Daltonics, Bremen, Germany). CD spectra were recorded on a JASCO J-810 spectropolarimeter.

X-ray diffraction data of λ,*R*_*a*_-**1**, δ,*S*_*a*_-**1**, and rac-**1**, were collected on a Bruker D8 Venture diffractometer using Mo Kα radiation (λ = 0.71073 Å). Single crystals were mounted and coated with perfluoropolyether oil. Diffracted intensities were integrated with SAINT,^58^ and absorption corrections were applied using the multi-scan strategy by SADABS,^59,60^ both implemented in the APEX5 software package. The structures were solved by direct methods using SHELXS^61^ and refined by full-matrix least-squares on F2 with SHELXL,^62^ within the WinGX system.^63^

#### Crystal Data for λ,*R*_*a*_-**1**

C_20_H_16_Cl_2_N_2_Pt, *M*_r_ = 550.34, yellow block, monoclinic, *C2*, *a* = 18.7708(6) Å, *b* = 7.6559(2) Å, *c* = 12.6900(4) Å, *β* = 100.3709(11)°, *V* = 1793.85(9) Å^3^, *Z* = 4, *T* = 100(2) K, *D*_calcd_ = 2.038 g cm^–3^, *μ* = 8.124 mm^–1^, absorption correction factors min. 0.491 max. 0.614, 36098 reflections, 4424 unique (*R*_int_ = 0.0336), 4413 observed, *R*_1_ = 0.0219 [I > 2σ­(I)], w*R*_2_(*F* ^2^) = 0.0562 (all data), GOF = 1.121. CCDC 2565453.

#### Crystal Data for δ,*S*_*a*_-**1**

C_20_H_16_Cl_2_N_2_Pt, *M*_r_ = 550.34, yellow block, monoclinic, *C2*, *a* = 18.8431(7) Å, *b* = 7.6646(3) Å, *c* = 12.6808(5) Å, *β* = 100.2641(11)°, *V* = 1802.11(12) Å^3^, *Z* = 4, *T* = 100(2) K, *D*_calcd_ = 2.028 g cm^–3^, *μ* = 8.087 mm^–1^, absorption correction factors min. 0.397 max. 0.738, 35017 reflections, 4484 unique (*R*_int_ = 0.0347), 4477 observed, *R*_1_ = 0.0257 [I > 2σ­(I)], w*R*_2_(*F* ^2^) = 0.0610 (all data), GOF = 1.092. CCDC 2565454.

#### Crystal Data for Rac-**1**

C_20_H_16_Cl_2_N_2_Pt, *M*_r_ = 550.34, yellow block, monoclinic, *P21*/*c*, *a* = 10.4868(5) Å, *b* = 10.5927(4) Å, *c* = 15.5697(7) Å, *β* = 96.6022(18)°, *V* = 1718.07(13) Å^3^, *Z* = 4, *T* = 100(2) K, *D*_calcd_ = 2.128 g cm^–3^, *μ* = 8.483 mm^–1^, absorption correction factors min. 0.559 max. 0.712, 71189 reflections, 4272 unique (*R*_int_ = 0.0491), 3938 observed, *R*_1_ = 0.0280 [I > 2σ­(I)], w*R*_2_(*F* ^2^) = 0.0680 (all data), GOF = 1.103. CCDC 2565455.

### General Procedure for the Synthesis of *cis*-[PtCl_2_(DABN)] Complexes

The corresponding DABN ligand was dissolved in CH_3_CN, and K_2_PtCl_4_ was added. The reaction mixture was stirred at 50 °C for 2 days, affording a yellow solution containing a suspended white solid, attributed to KCl. After cooling to room temperature, 5 mL cold water was added, which dissolved KCl and, in all cases, induced precipitation of the product as a yellow solid. It was then collected by filtration and dried under reduced pressure. Yellow crystals suitable for single-crystal X-ray diffraction were obtained in all cases by slow vapor diffusion of Et_2_O into a saturated CH_3_CN solution of the corresponding complex at 4 °C.

### Synthesis of λ,*R*_*a*_-**1**

Following the general procedure, *R*_a_-DABN (170.80 mg, 0.60 mmol), K_2_PtCl_4_ (250.18 mg, 0.60 mmol) were reacted in CH_3_CN (10 mL) affording λ,*R*_*a*_ **-1** as a yellow solid. Yield: 87% (287.68 mg, 0.52 mmol).

### Synthesis of δ,*S*_*a*_-**1**

Following the general procedure, *S*_a_-DABN (172.27 mg, 0.61 mmol), K_2_PtCl_4_ (252.41 mg, 0.61 mmol) were reacted in CH_3_CN (10 mL) affording δ,*S*_*a*_-**1** as a yellow solid. Yield: 85% (284.21 mg, 0.52 mmol).

### Synthesis of Rac-**1**

Following the general procedure, rac-DABN (42.97 mg, 0.15 mmol), K_2_PtCl_4_ (62.89 mg, 0.15 mmol) were reacted in CH_3_CN (5 mL) affording rac-**1** as a yellow solid. Yield: 60% (48.75 mg, 0.09 mmol).

### Spectroscopic Data

λ,*R*_*a*_-**1**, δ,*S*_*a*_-**1**, and rac-**1** were obtained as yellow solids. All compounds are insoluble in water and soluble in CD_3_CN. They are initially soluble in DMSO, although DMSO promotes ligand exchange over time (see main text). All three samples showed identical NMR and HRMS data. ^1^H NMR (300 MHz, CD_3_CN, 298 K): δ (ppm) 8.16 (d, ^3^ *J*_H–H_ = 8.7 Hz, 2H, H4), 8.06 (d, ^3^ *J*_H–H_ = 8.2 Hz, 2H, H6), 7.58 (d, ^3^ *J*_H–H_ = 8.8 Hz, 2H, H3), 7.52 (t, ^3^ *J*_H–H_ = 8.2 Hz, 2H, H7), 7.35 (t, ^3^ *J*_H–H_ = 8.3 Hz, 2H, H8), 7.03 (d, ^3^ *J*_H–H_ = 8.5 Hz, 2H, H9), 5.85–5.54 (br, 4H, NH_2_). ^13^C­{^1^H} NMR (101 MHz, CD_3_CN, 298 K): δ 138.5 (C2), 134.7 (C10), 133.2 (C5), 132.2 (C4), 129.5 (C6), 128.3 (C8), 126.8 (C7), 126.3 (C9), 122.0 (C1), 121.1 (C3). ^195^Pt­{^1^H} NMR (121 MHz, CD_3_CN, 298 K) δ −1914.4 (br, Pt). HRMS (ESI+, DMF): m/z calcd. for C_20_H_16_Cl_2_N_2_PtNa [**1** + Na]^+^, 572.0232; found, 572.0239.

## Supplementary Material



## Notes

Multinuclear NMR; HRMS; CD spectra; and X-ray data for all compounds (PDF)
