# Supplementary material for: Diastereoselective Axial-to-Chelate-Puckering Chirality Transfer in cis-[PtCl2(DABN)] Complexes
Source: Inorg Chem. 2026 Sep 9;65(42):24745–52. doi: 10.1021/acs.inorgchem.6c03354 (PMC13715576; doi:10.1021/acs.inorgchem.6c03354)
Supplement: Supplementary file 1 [file ic6c03354_si_001.pdf]

SUPPORTING INFORMATION

Diastereoselective Axial-to-Chelate-Puckering Chirality Transfer in *cis*-[$PtCl_2$(DABN)] Complexes

Andrea Maluenda,[a] Tamara Bolaño,[b,*] Pablo J. Sanz Miguel [a,*]

[a] Departamento de Química Inorgánica, Instituto de Síntesis Química y Catálisis Homogénea (ISQCH), Universidad de Zaragoza-CSIC. 50009, Zaragoza, Spain.

[b] Departamento de Química Inorgánica, Instituto de Nanociencia y Materiales de Aragón (INMA), Universidad de Zaragoza-CSIC. 50009, Zaragoza, Spain.

* Email addresses: tbg@unizar.es (TB); pablo.sanz@unizar.es (PJSM)

Contents

NMR spectra S2

HRMS spectra S7

CD spectra S8

X-ray data S9

NMR spectra

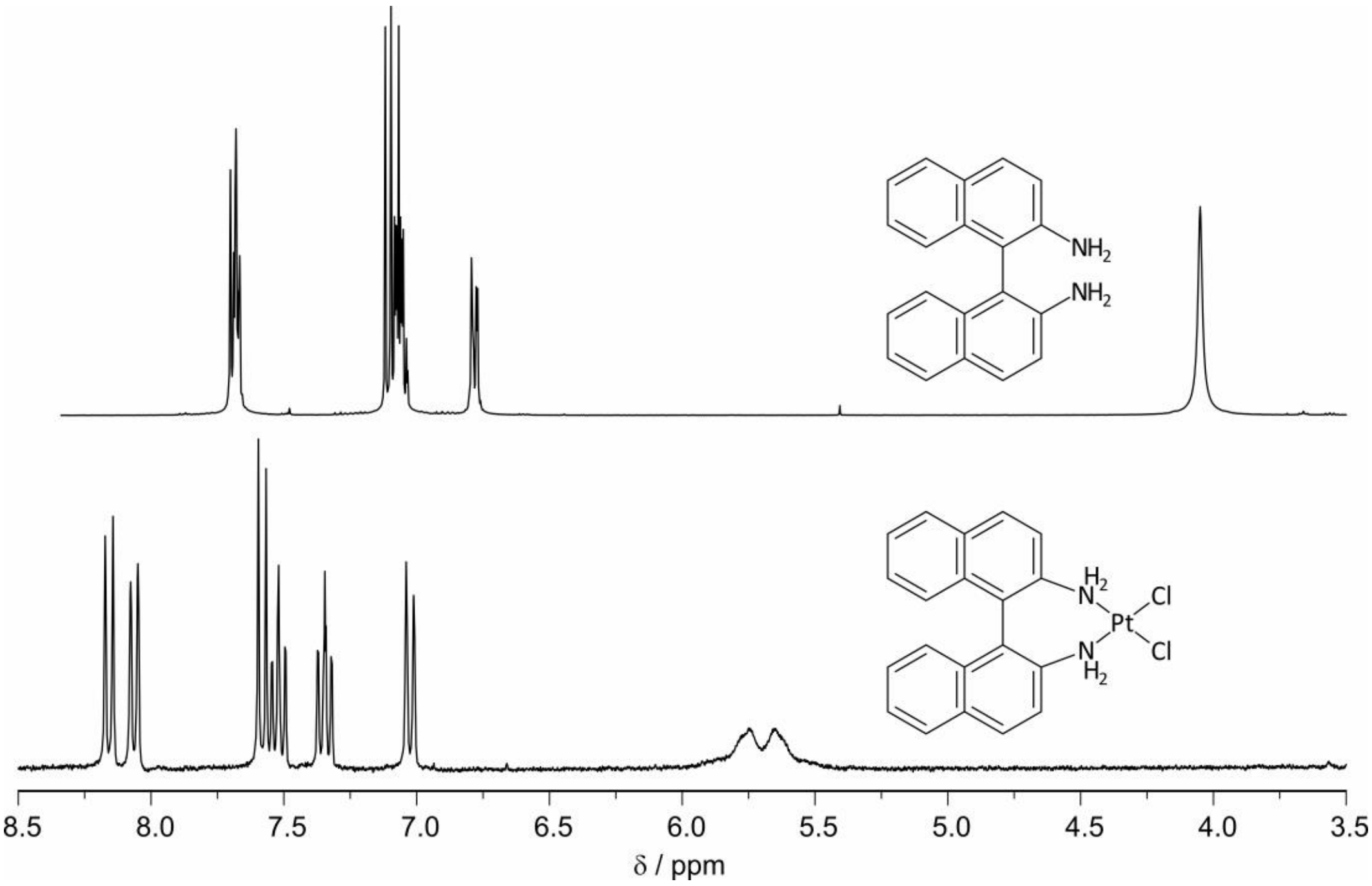

Figure S1. Stack plot of ^{1}H NMR spectra (400 MHz, CD_3CN, 298 K) of commercial DABN (up), and λ,R_a-**1** (bottom).

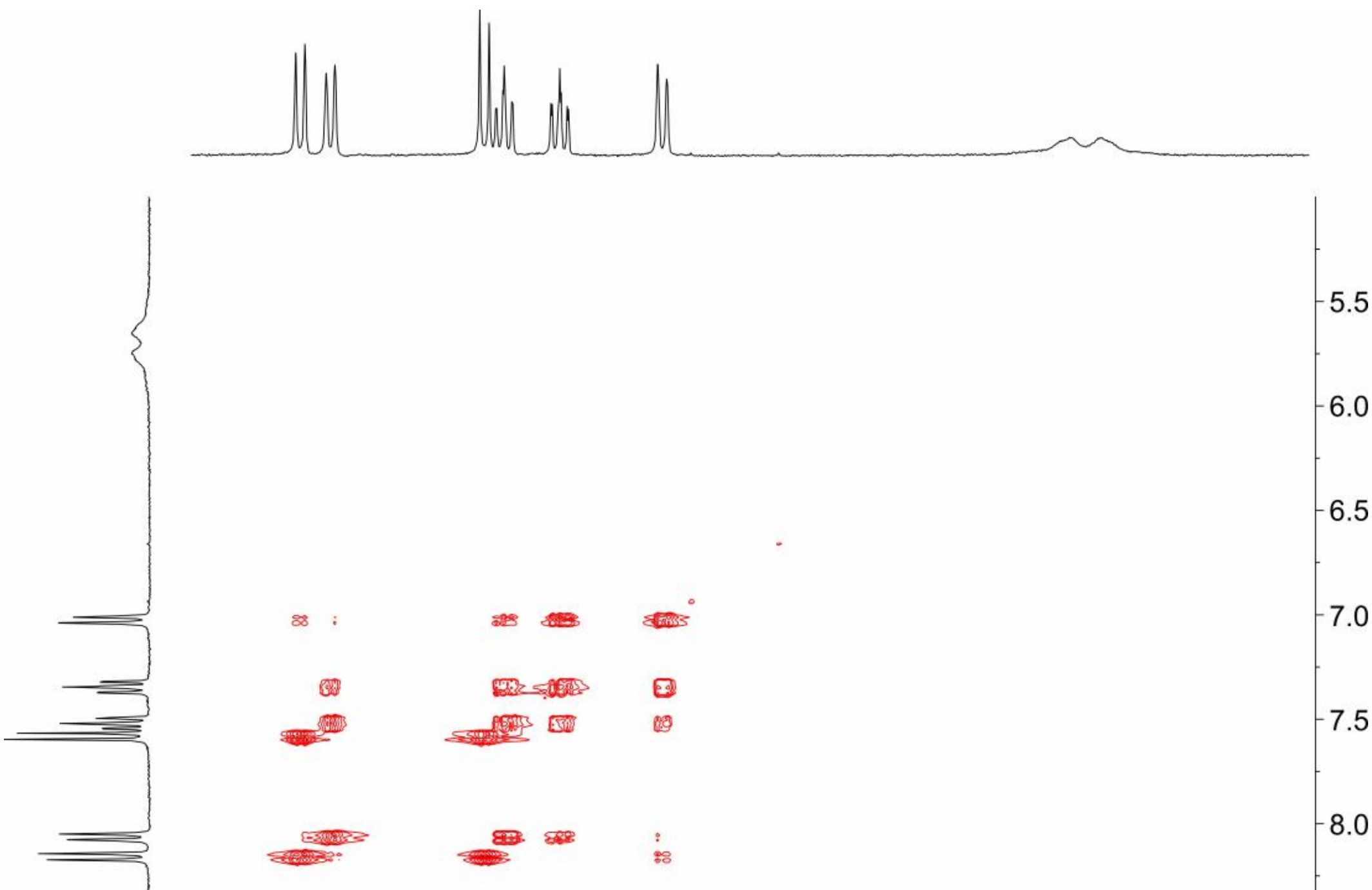

Figure S2. ^{1}H-^{1}H COSY NMR spectrum of λ,R_a-**1** (300 MHz, CD_3CN, 298 K).

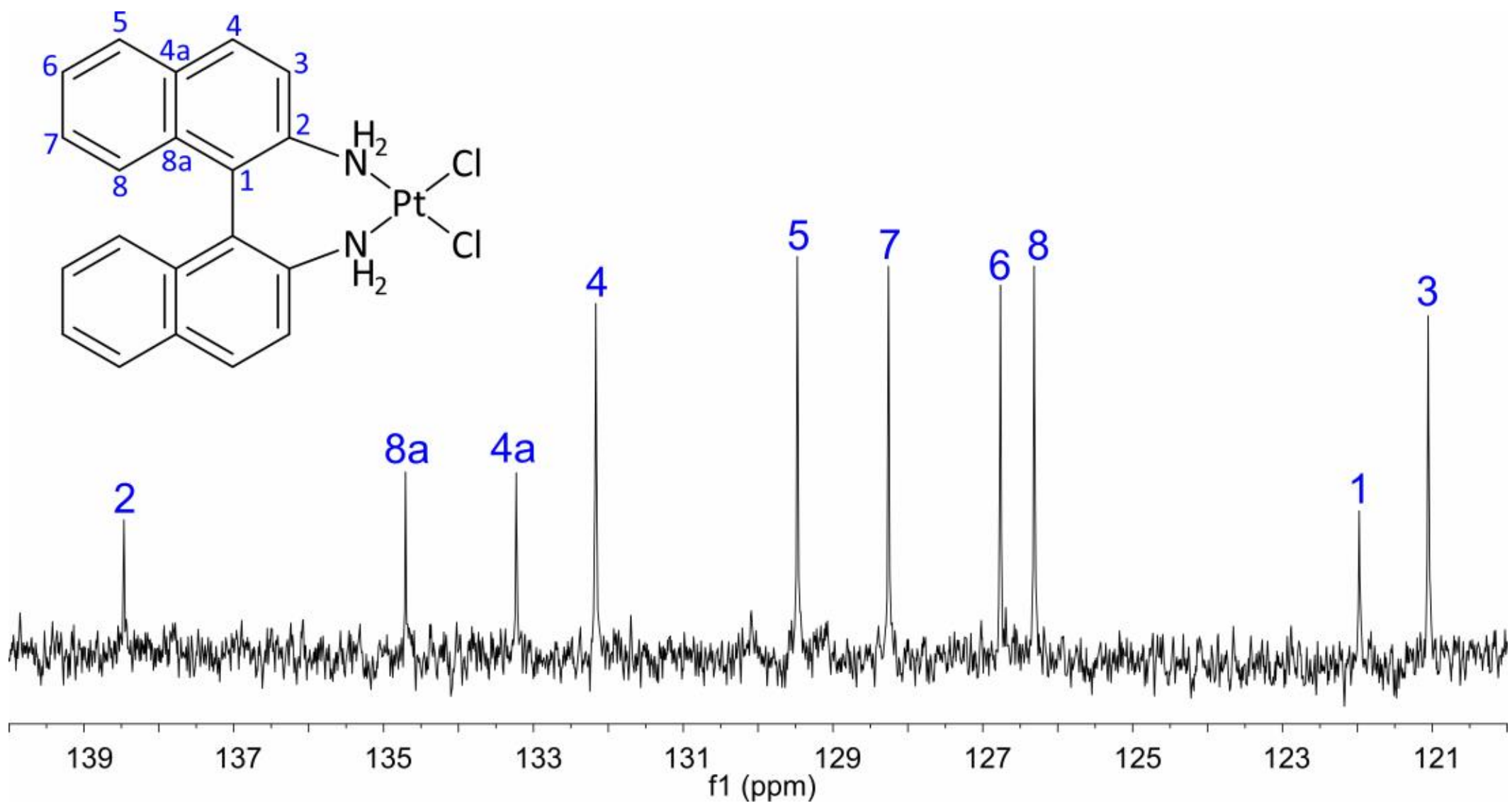

Figure S3. $^{13}C\{^{1}H\}$ NMR spectrum of λ,R_a-**1** (300 MHz, CD_3CN, 298 K).

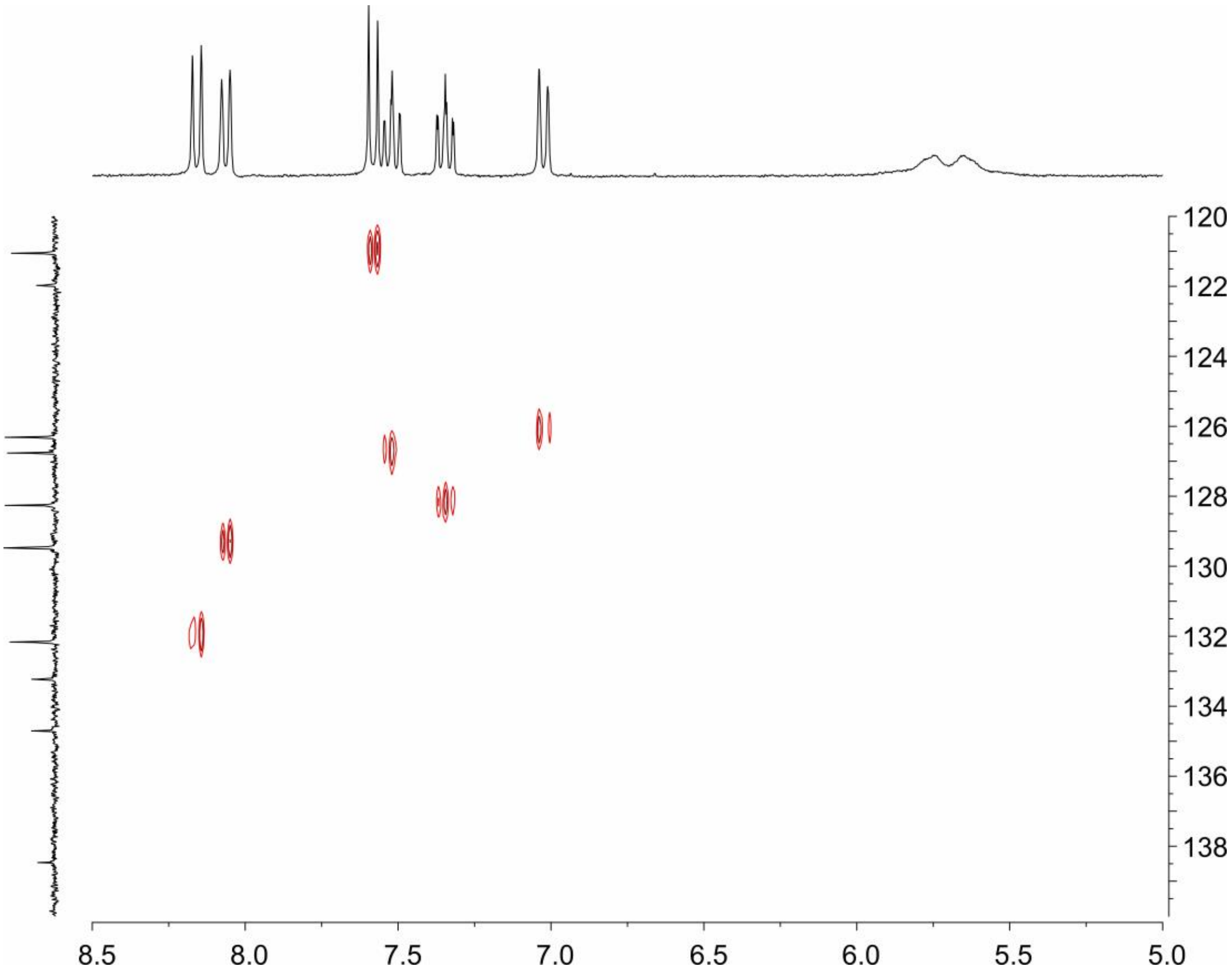

Figure S4. ^{1}H-^{13}C HSQC NMR spectrum of λ,R_a-**1** (300 MHz, CD_3CN, 298 K).

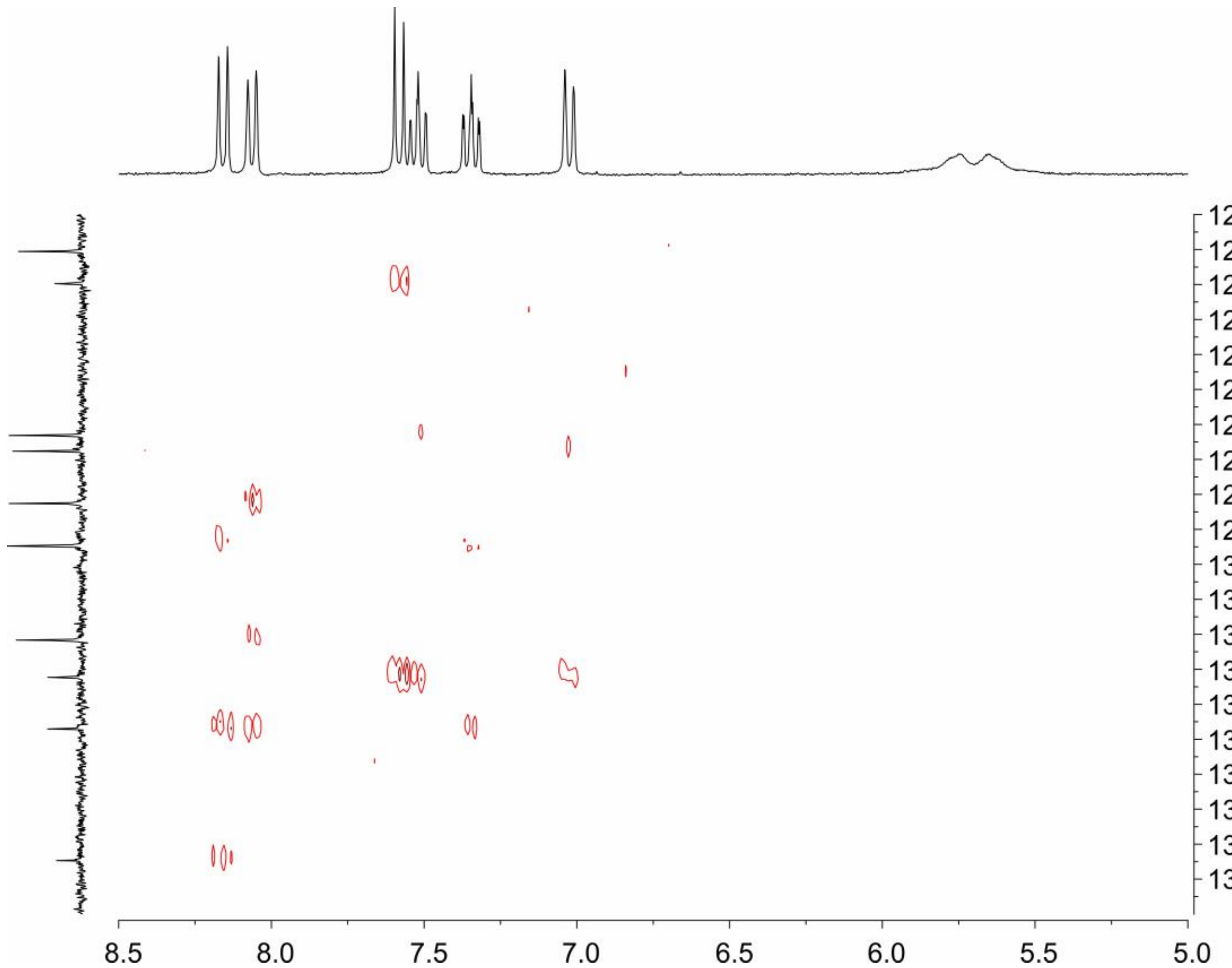

Figure S5. ^1H-^{13}C HMBC NMR spectrum of λ,R_a-**1** (300 MHz, CD_3CN, 298 K).

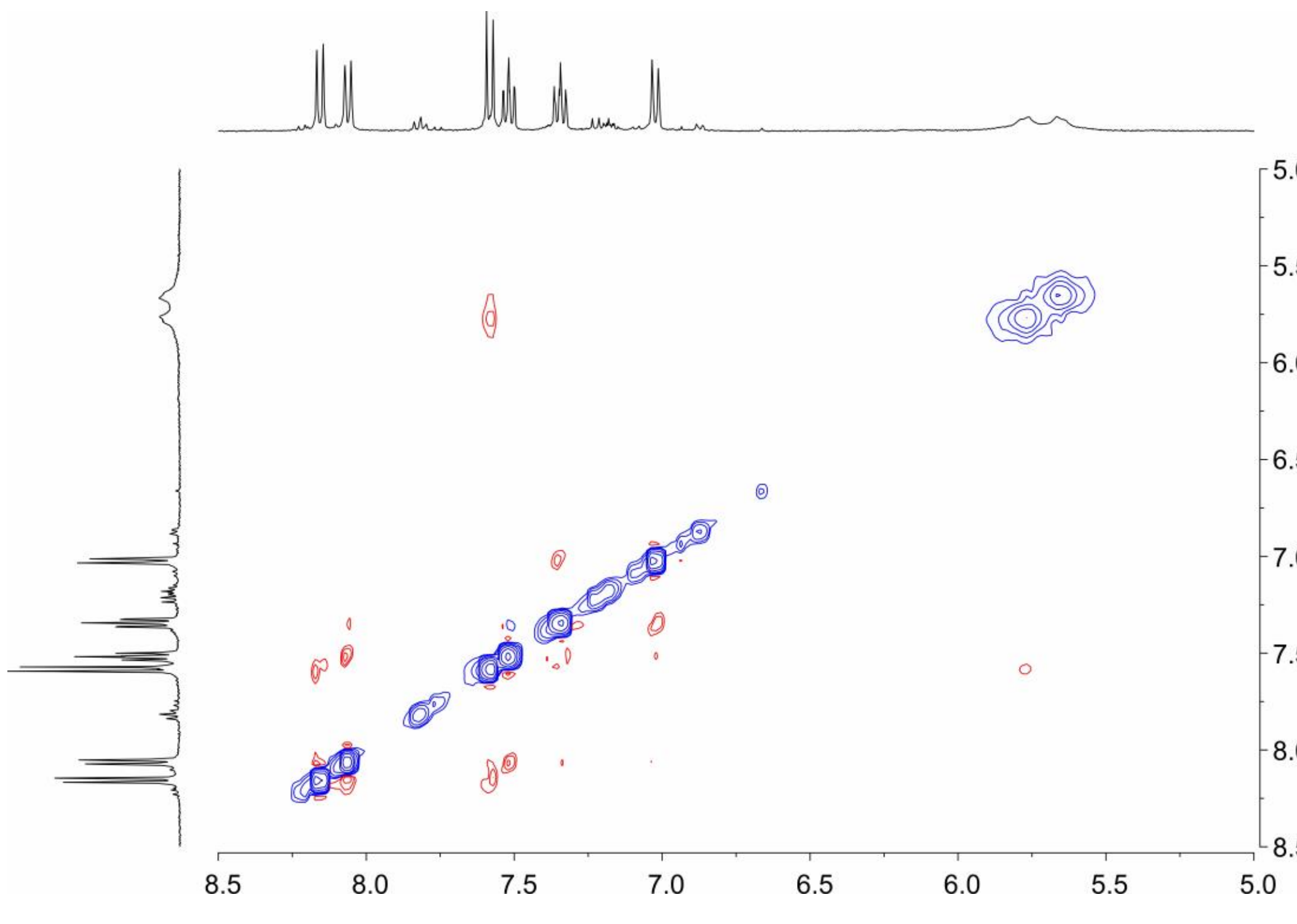

Figure S6. ^1H-^1H NOESY NMR spectrum of λ,R_a-**1** (300 MHz, CD_3CN, 298 K).

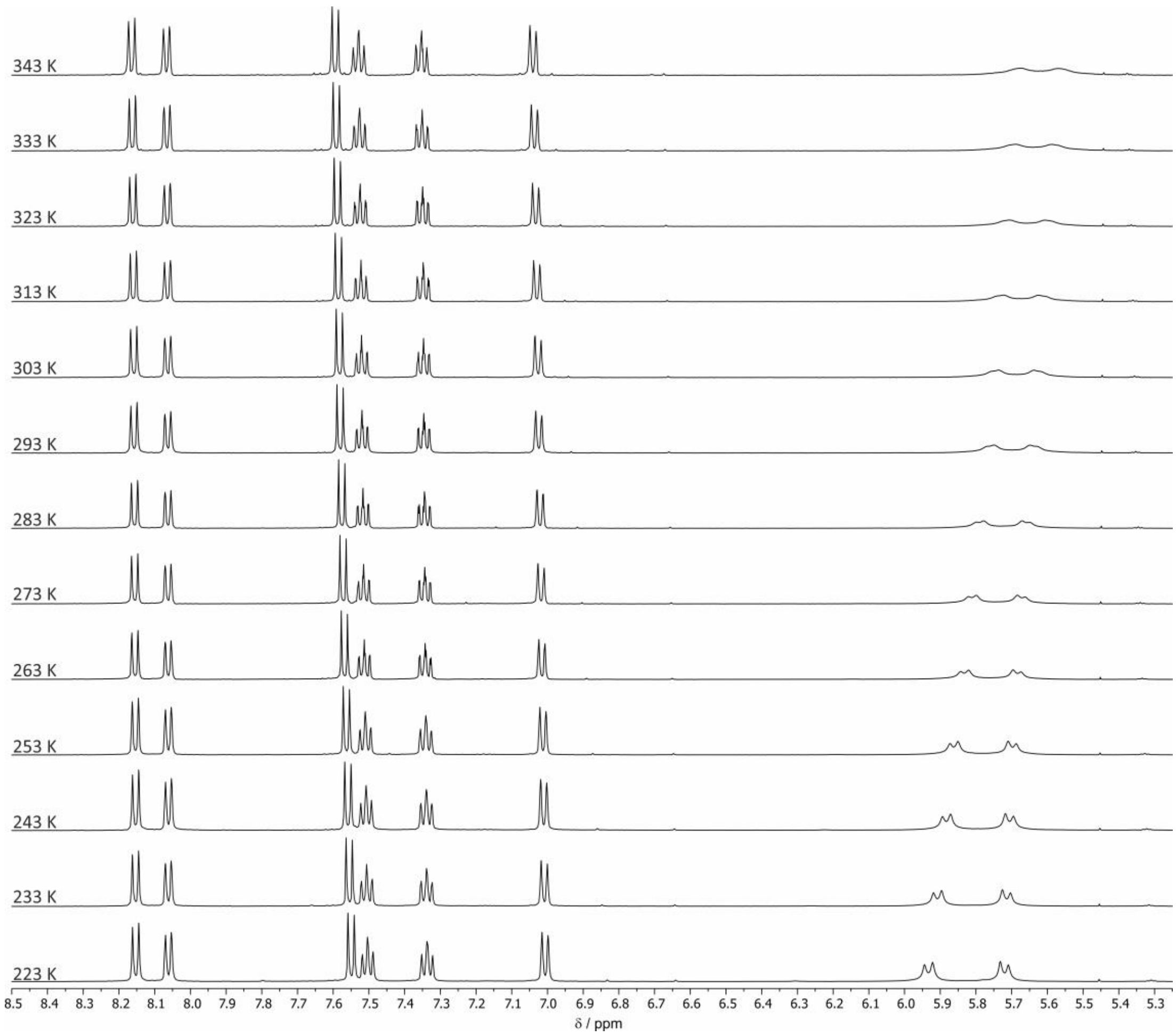

Figure S7. Variable temperature ^{1}H NMR spectra of λ,R_a-**1** recorded at 500 MHz in CD_3CN.

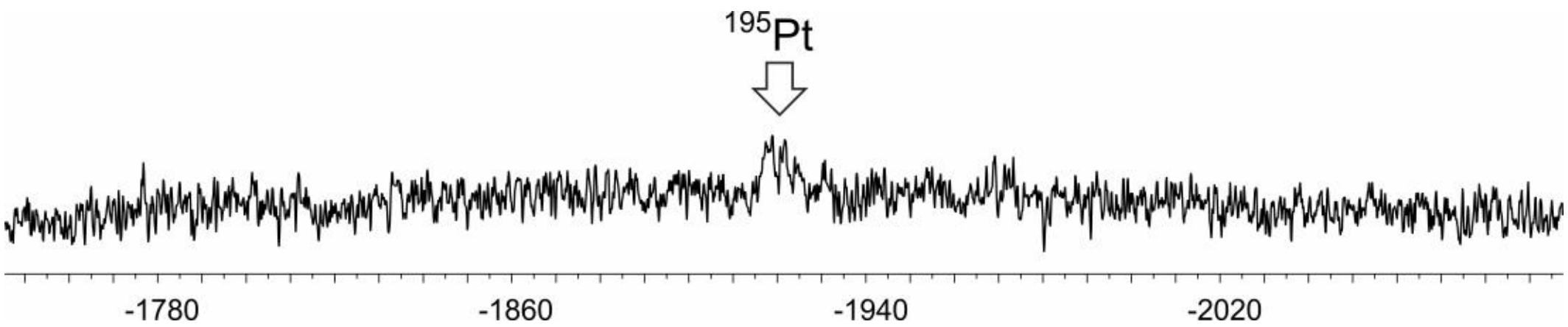

Figure S8. ^{195}Pt{^{1}H} NMR spectrum of λ,R_a-**1** (300 MHz, CD_3CN, 298 K).

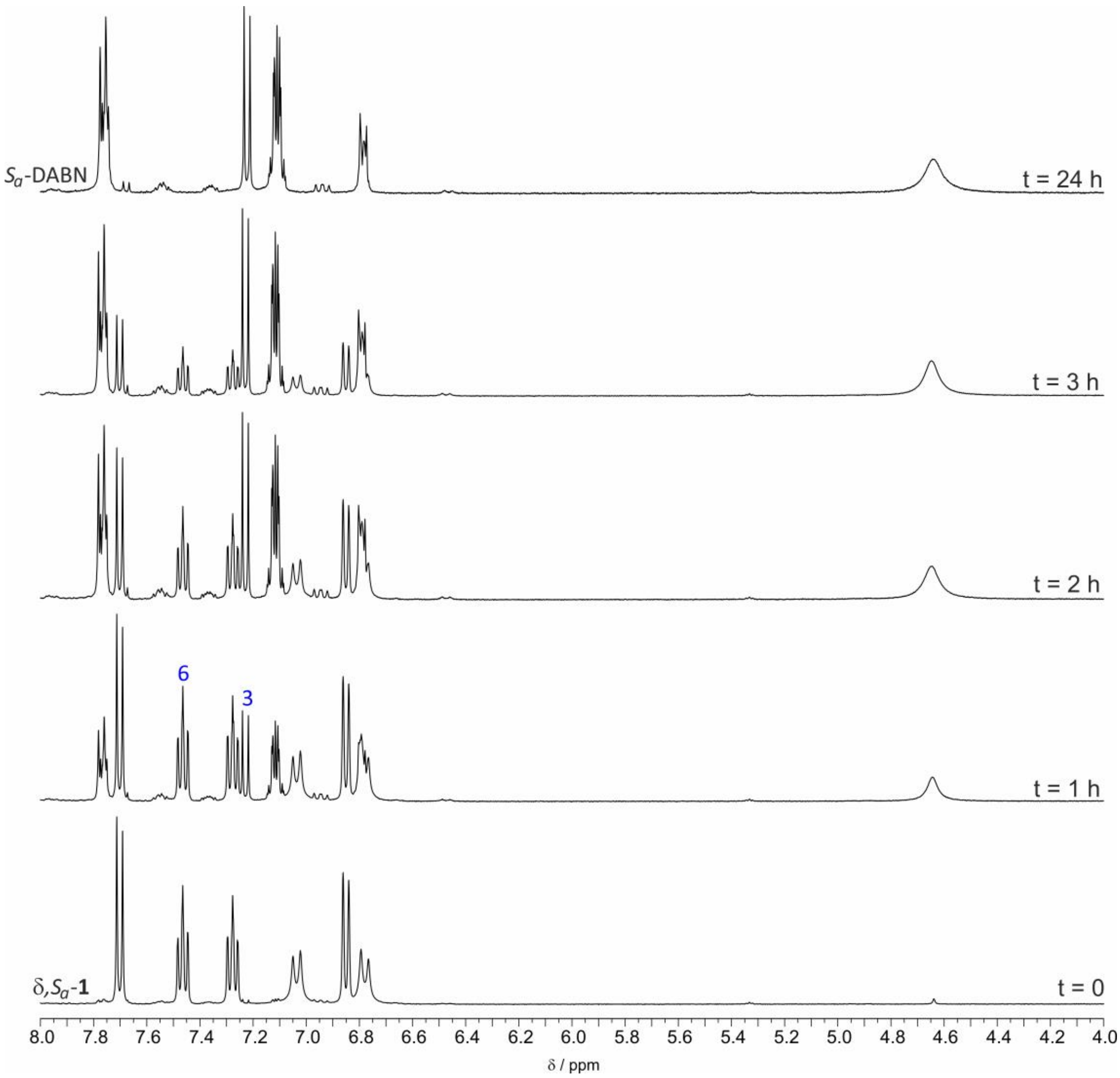

Figure S9. Time-dependent ^{1}H NMR of δ,S_a-**1** by (DMSO-d_6, r.t.), showing displacement of coordinated DABN and the appearance of the NH_2 resonances of free S_a-DABN.

Table S1. Kinetic fits for the degradation of δ,S_a-**1** (left) and the release of free DABN (right).

	Degradation of δ,S_a-**1**	Release of DABN
Equation	$y = A_1*\exp(-x/t_1) + y_0$	$y = A_1*\exp(-x/t1) + y_0$
y_0	0.06579 ± 0.00456	0.93421 ± 0.00456
A_1	0.97531 ± 0.0045	–0.97531 ± 0.0045
t_1	157.02793 ± 1.78448	157.02793 ± 1.78448
Reduced Chi-Sqr	4.09898 10^{-5}	4.09898 10^{-5}
R-Square (COD)	0.99926	0.99926
Adj. R-Square	0.99923	0.99923

HRMS spectra

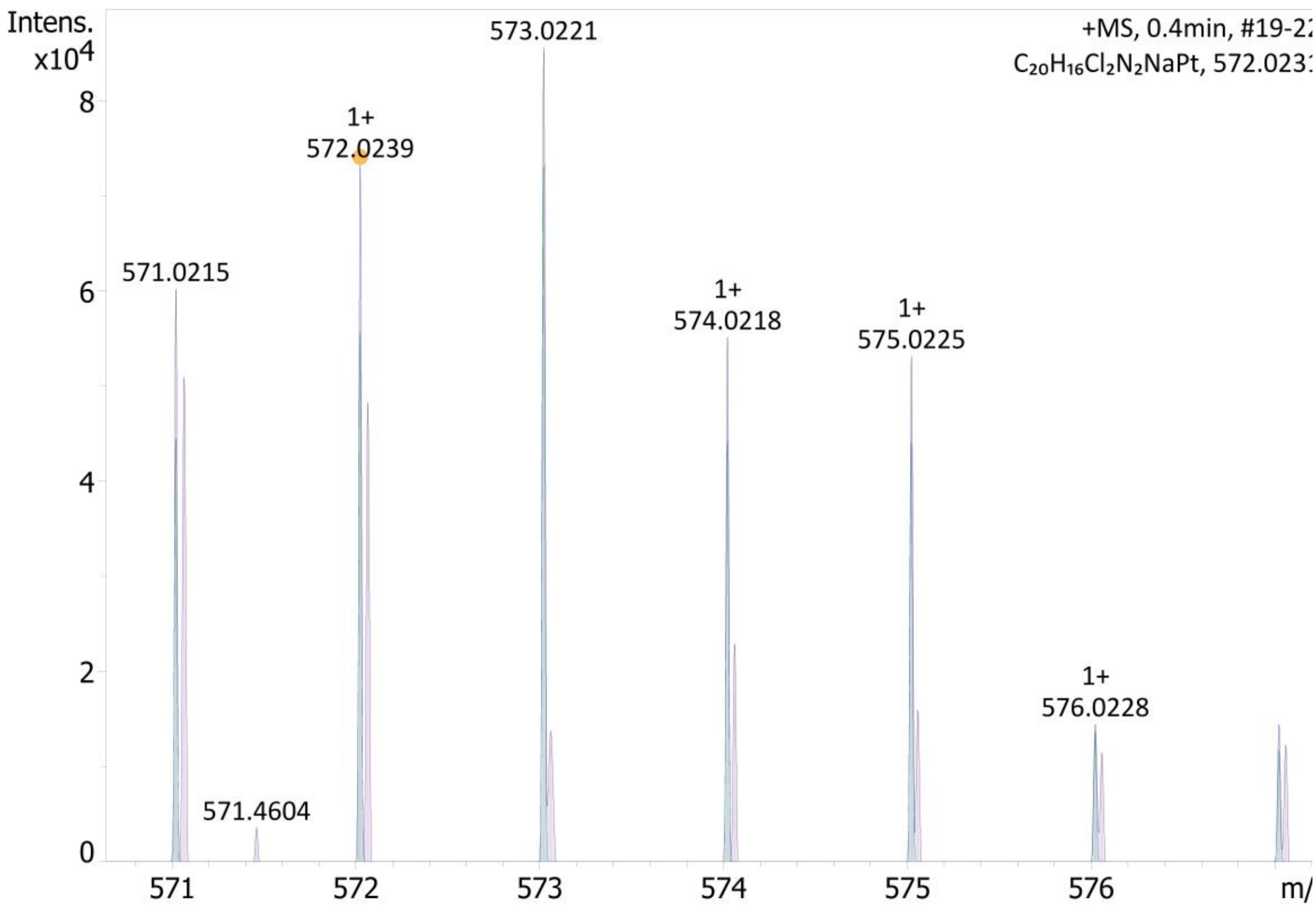

Figure S10. HRMS spectrum of λ,R_a-**1** (DMF, ESI(+), m/z, 298 K). Calculated isotopic pattern in green and experimental spectrum in purple.

CD spectra

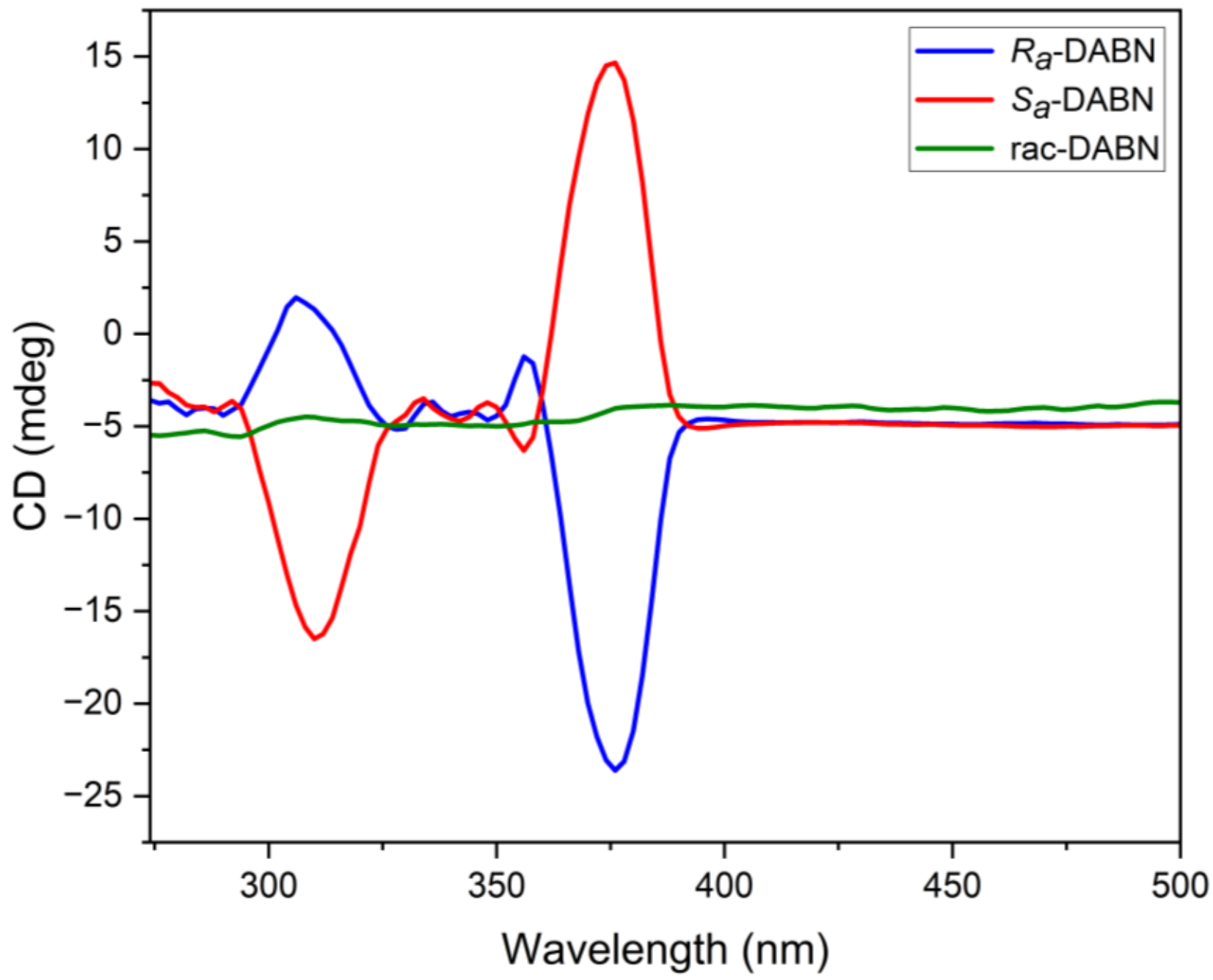

Figure S11. CD spectra of the DABN atropisomers R_a-DABN and S_a-DABN, together with their racemic mixture rac-DABN.

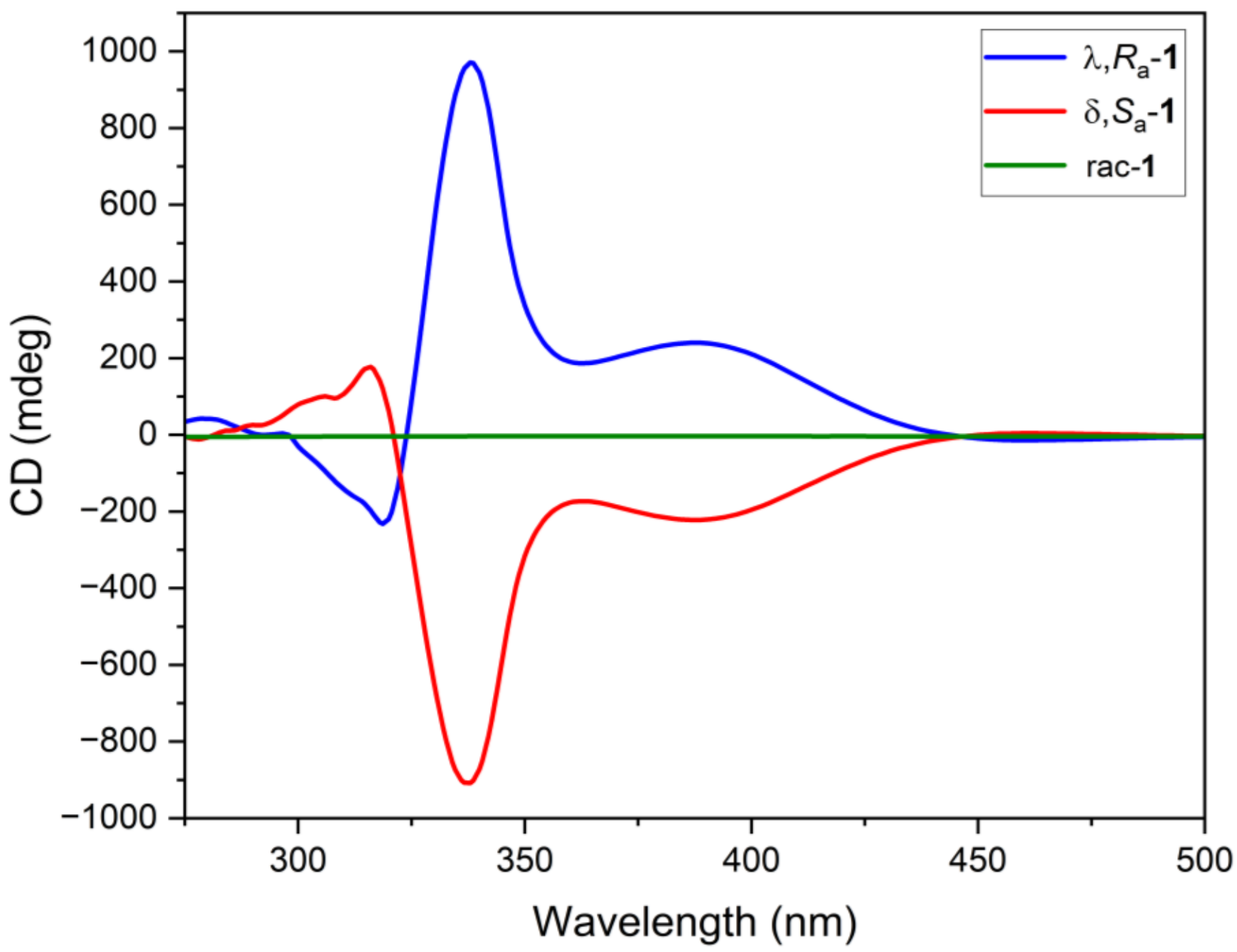

Figure S12. CD spectra of λ,R_a-**1** y δ,S_a-**1** and rac-**1**.

X-ray data

Table S1. Selected bond lengths [Å] and angles [°] in the crystal structures of compound **1**.

	λ,R_a-**1**	δ,S_a-**1**	rac-**1**
Pt–N2	2.051(6)	2.050(7)	2.055(3)
Pt–N12	2.069(5)	2.072(6)	2.067(3)
Pt–Cl1	2.2934(17)	2.292(2)	2.3027(10)
Pt–Cl2	2.3001(17)	2.300(2)	2.3000(10)
N2–C2	1.452(8)	1.448(9)	1.448(9)
N12–C12	1.457(7)	1.454(9)	1.454(9)
C1–C2	1.375(8)	1.381(9)	1.378(6)
C11–C12	1.386(8)	1.384(9)	1.385(6)
C1–C11	1.498(7)	1.496(8)	1.492(5)
N2–Pt–N12	90.57(19)	90.3(2)	91.35(13)
N2–Pt–Cl1	177.90(18)	178.0(2)	176.90(10)
N2–Pt–Cl2	86.16(15)	86.44(18)	87.72(10)
N12–Pt–Cl1	89.06(14)	89.37(17)	87.06(10)
N12–Pt–Cl2	176.32(15)	176.16(19)	177.19(10)
Cl1–Pt–Cl2	94.27(7)	94.00(10)	94.00(4)
C2–N2–Pt	114.9(4)	114.8(5)	111.4(2)
C12–N12–Pt	110.6(4)	111.2(4)	114.7(2)
N2–C2–C1	119.7(5)	119.6(6)	118.9(4)
N12–C12–C11	120.5(5)	119.9(6)	120.3(4)
C1–C11–C12	119.2(5)	119.7(6)	120.4(4)
C11–C1–C2	119.1(5)	118.9(6)	118.9(4)
